# Shielding effect of a lead apron on the peripheral radiation dose outside the applicator of electron beams from an Elekta linear accelerator

**DOI:** 10.1002/acm2.13089

**Published:** 2020-12-09

**Authors:** Huifang He, Yingdong Zhang, Jidong Wang, Xingyu Chen, Yuyan Yang, Junjie Wang

**Affiliations:** ^1^ Department of Radiation Oncology Peking University International Hospital Beijing China; ^2^ Department of Radiation Oncology Peking University 3rd Hospital Beijing China

**Keywords:** electron beams, out‐of‐field radiation, shielding effect of LA

## Abstract

**Purpose:**

To evaluate the shielding effect of lead aprons (LAs) on peripheral radiation doses outside the applicator of electron beams from a linear accelerator.

**Methods:**

Out‐of‐field radiation doses of 4‐, 6‐, 8‐, 10‐, 12‐, and 15‐MeV electron beams from an Elekta Synergy linear accelerator (linac) were measured by thermoluminescence dosimeters (TLD) at different depths (0, 0.5, 1.0, and 2.0 cm) and distances from the applicator edge (0–58 cm) in a water‐equivalent slab phantom with a different number of layers of LA shielding (0–5 layers). Measurements were performed by 6 × 6, 10 × 10, 14 × 14, and 20 × 20‐cm^2^ applicators at a gantry and collimator angle of 0°. The out‐of‐field radiation dose profiles were normalized to the maximum dose of every energy and measuring depth.

**Results:**

The out‐of‐field radiation doses (beyond 3 cm away from the field edge) decreased with an increase in the number of LA layers and distance away from the central beam axis (CAX). After shielding with the LA, the out‐of‐field doses decreased by up to approximately 99% compared with the no shielding group. For 4‐MeV electron beams, there was a peak at 24.5 cm from the CAX, which weakened with an increasing number of LA layers.

**Conclusion:**

The shielding effect of the LA varied for a different number of LA layers as well as different depths and distances away from the CAX. Four LA layers were sufficient for shielding out‐of‐field doses of 4–15‐MeV electron beams.

## 1 INTRODUCTION

Advances in radiotherapy have greatly improved the ability to deliver highly conformal doses to tumors while minimizing doses to surrounding normal tissues. Despite this tremendous progress, there remains a general concern about out‐of‐field radiation. Since the beginning of the 1950s, megavoltage electron beams have been used as an important treatment modality in modern radiotherapy owing to the advantage of a sharp dose drop to off beyond the tumor site. Electron therapy has often been used in the treatment of superficial tumors (i.e., at depths <5 cm), and the selection of energy levels (range 4–20 MeV) mainly depends on the tumor invasion depth. The dimension of the treatment field for electron beams is defined by an applicator, and radiation doses outside the treatment field are associated with scattered radiation and leakage radiation from the electron applicators and treatment gantry. An ideal electron applicator should remove all electrons outside the treatment field. In terms of electron therapy, out‐of‐field radiation is generally negligible unless it is very close to organs at risk (OARs), such as the lens. However, current treatment planning systems (TPSs) are not designed for the calculation of out‐of‐field radiation doses due to a poor calculation accuracy.

Out‐of‐field radiation is detrimental to patients, in particular children, pregnant patients, and patients with implanted electronic devices.^1^ Furthermore, in the post‐treatment period, late effects, and secondary cancers induced by radiation may manifest in survivors.^2^, ^3^, ^4^ Thus, out‐of‐field radiation should not be ignored. Recently, Cardenas et al. reported that an appropriate shielding strategy was very important for patients and that effective body protection measures were clinically significant.^5^ However, the shielding effect of a lead apron (LA) on patient protection cannot be fully assessed using modern TPSs. In this paper, quantifying the shielding effect of LAs on out‐of‐field dose distributions was performed for electron beams from a linear accelerator, as this has not been reported in the literature. A satisfactory shielding effect with LAs was observed.

## 2 MATERIALS AND METHODS

The Elekta Synergy linear accelerator (Crawley, UK) used in this study was fitted with a multileaf collimator with 60 leaves that automatically opened to a specified size whenever the electron energy was selected and the electron applicator was attached to the accessory mount within the gantry head. There were six electron beam energy levels associated with the accelerator: 4, 6, 8, 10, 12, and 15 MeV, and the irradiation fields were defined by 6 × 6, 10 × 10, 14 × 14, and 20 × 20‐cm^2^ applicators. The distance between the source within the gantry head and the end of the applicator was 95 cm. The linac was calibrated to deliver 1 cGy/MU beam under reference conditions (field size = 10 × 10 cm^2^, source‐to‐surface distance [SSD] = 100 cm) at the depth of maximum dose (Dmax).

Thermoluminescence dosimeters (TLDs; cylindrical LiF:Mg,Cu,P GR‐200B, Beijing Guangrun Yitong Radiation Monitoring Equipment Co. LTD) that were 4.5 mm in diameter and 0.8 mm in thickness were used, whose dispersity, linearity range, and dose range were 1%, 10^−7^–10 Gy, and 10 μGy–10 Gy (data provided by production company), respectively, and TLDs were calibrated before purchase, whose reading is consistent for all energies. The LiF: Mg,Cu,P TLDs were annealed before each irradiation at 240°C for 10 min with air circulation whose temperature was controlled within ±2°C. Then, the TLDs were removed and rapidly cooled with an air fan. Readings were performed with a GRYT series TLD RGD‐E reader. The readout program was as follows: heating rate of 20°C.s^−1^, preheating at 140°C for 20 s, readout between 140°C and 240°C, and hold 240°C for 20 s.

**FIG. 1:**
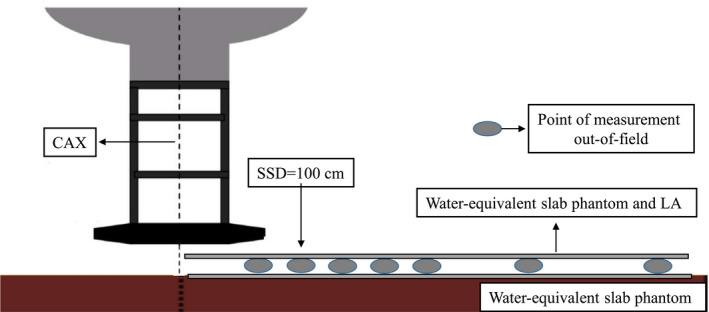
Schematic diagram of arrangement of the thermoluminescence dosimeters in the solid water phantom to measure peripheral dose outside the applicator.

Doses outside of the 10 × 10‐cm^2^ applicator field were measured for electron beams at 4, 6, 8, 10, 12, and 15 MeV at least three times, and 500 MU were delivered for each measurement. Peripheral doses of the 6 × 6, 14 × 14, and 20 × 20‐cm^2^ applicators were also measured for 6 and 12 MeV at a depth of 0.5 cm. The gantry and collimator were set to 0º for all measurements. The TLDs were positioned on the surface of a water‐equivalent slab phantom (SP34, IBA Dosimetry, Schwarzenbruck, Germany.) with the center axis parallel to the line from the gun to the target position, with the gantry at SSD = 100 cm and at 0, 0.5, 1.0, and 2.0‐cm depths of the phantom and distances from the field edge (0–58 cm), respectively (see Fig. 1). All measurements were performed at SSD = 100 cm; thus, there was a 5‐cm gap between the applicator end and solid water phantom surface with the gantry at 0°. A different number of layers of an LA (0.5 mm for each layer) were applied to cover the surface of the TLDs and phantom. For electron beams with different energy levels and measuring depths, the out‐of‐field TLD readings were normalized to the dose (Dref) measured at the following conditions: 10 × 10 cm^2^ applicator, 6 MeV, 0.5‐cm depth, no LA, and 5 cm away from the central beam axis (CAX).

## 3 RESULTS

### 3.A Shielding effect at 0‐cm depth

**FIG. 2:**
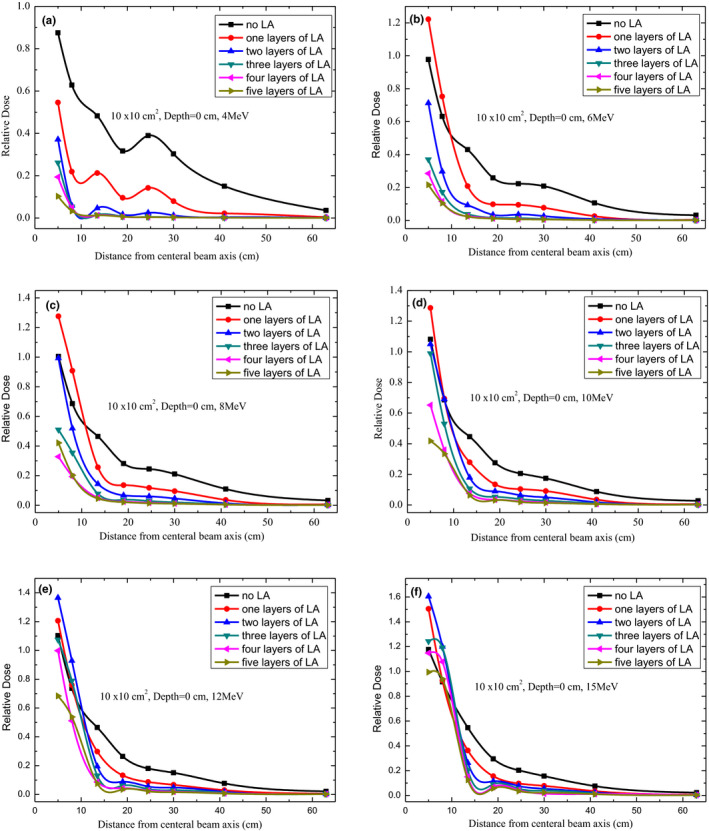
Out‐of‐field dose profiles for 4, 6, 8, 10, 12, and 15 MeV at a depth of 0 cm under different layers of lead aprons using 10 cm × 10 cm^2^ applicator.

Figure 2 displays the results of out‐of‐field dose variations before and after shielding with a different number of layers of an LA according to the distance from the beam CAX for a 10 × 10‐cm^2^ applicator at a 0‐cm depth. For 4‐MeV electron beams, the out‐of‐field dose naturally decreased as the number of layers of the LA increased. At four layers of the LA, the out‐of‐field dose decreased from 48.2% to 1.3% at 13.5 cm from the CAX, as illustrated in Fig. 2(a). However, at five layers of the LA, the out‐of‐field dose displayed no obvious change. For less than three LA layers, there was a peak at 24.5 cm from the CAX, which weakened with an increase in the number of LA layers and reached 39.0%, 14.2%, and 2.5%, respectively. Beyond the peak, the out‐of‐field dose decreased as a function of the distance from the CAX.

**FIG. 3:**
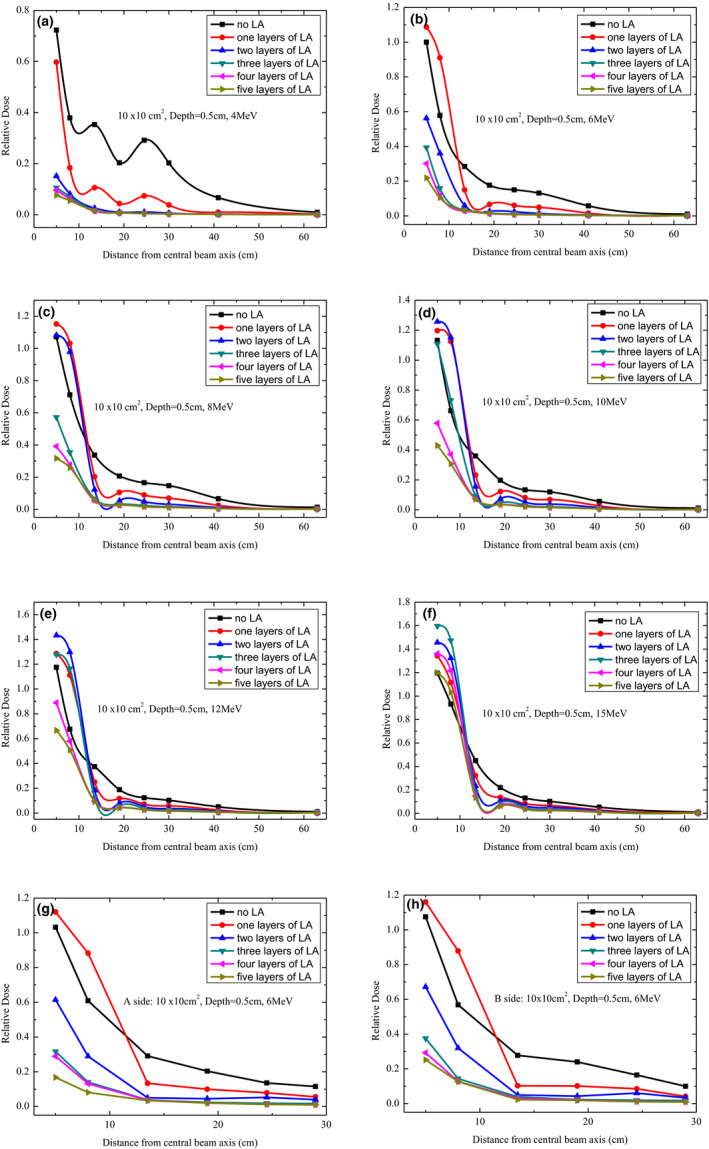
Out‐of‐field dose profiles for 4, 6, 8, 10, 12, and 15 MeV at a depth of 0.5 cm under different layers of lead aprons using 10 cm × 10 cm^2^ applicator.

**FIG. 4:**
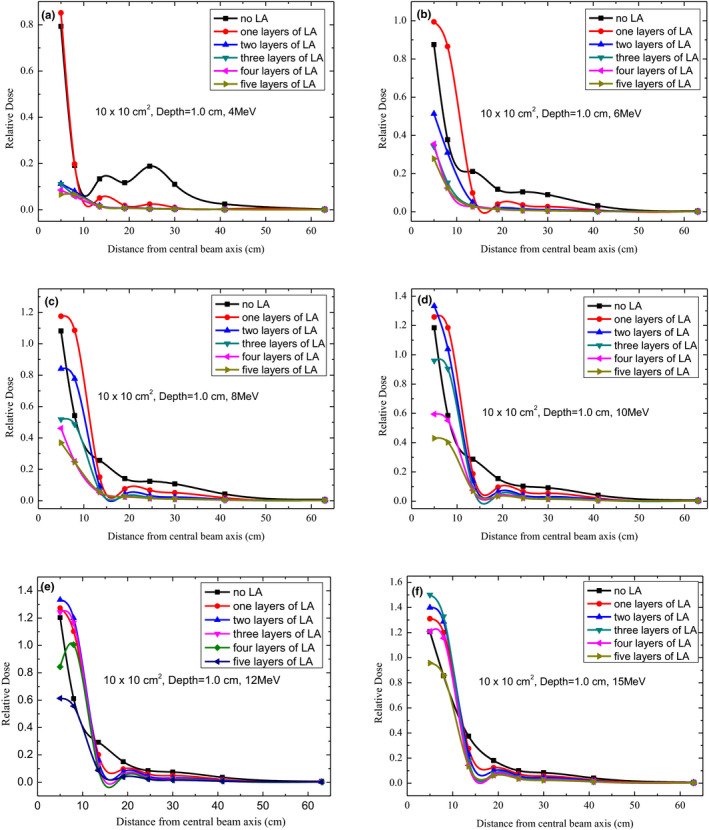
Out‐of‐field dose profiles for 4, 6, 8, 10, 12, and 15 MeV at a depth of 1.0 cm under different layers of lead aprons using 10 cm × 10 cm^2^ applicator.

**FIG. 5:**
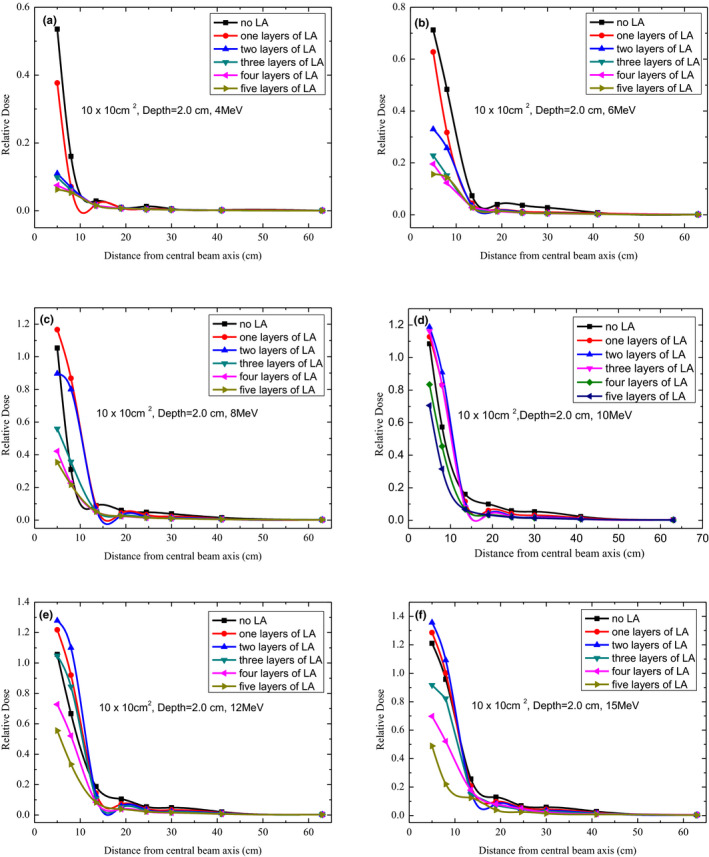
Out‐of‐field dose profiles for 4, 6, 8, 10, 12, and 15 MeV at a depth of 2.0 cm under different layers of lead aprons using 10 cm × 10 cm^2^ applicator.

A similar phenomenon was also observed at the same distance from the CAX at 0.5, 1.0, and 2.0‐cm depth for a 4‐MeV electron beam (Figs. 3, 4, 5). For 6‐MeV electron beams, as illustrated in Fig. 2(b), the out‐of‐field doses also generally decreased with an increase in the number of LA layers. However, at 5 and 8 cm from the CAX, a higher out‐of‐field dose was observed for a one‐layer LA than no LA, which may have been caused by the dose buildup effect. Moreover, for 8‐, 10‐, 12‐, and 15‐MeV electron beams, as illustrated in Figs. 2(c)–2(f), the dose buildup effect was also observed at a 5 and 8 cm distance from the CAX. A similar phenomenon was also observed at 0.5, 1.0, and 2.0‐cm depth (Figs. 3, 4, 5). Thus, a significantly different shielding effect of the LA was observed on out‐of‐field doses for different beam energies. Comparing a five‐layer LA with no LA at a 30‐cm distance from the CAX, the decrease in the out‐of‐field dose was 99.0%, 97.4%, 94.9%, 92.3%, 89.7%, and 85.4% for 4, 6, 8, 10, 12, and 15 MeV, respectively. Therefore, the shielding effect of the LA on the out‐of‐field dose decreased with the increase in beam energy at a 0‐cm depth. The shielding effect of the LA on the peripheral doses also gradually weakened with an increase in the distance from the CAX.

### 3.B Shielding effect at 0.5‐cm depth

Figure 3 illustrates the out‐of‐field relative doses as a function of the distance from the CAX for different energy levels and different LA layers at a 0.5‐cm depth of a solid water slab. We observed that for all energy levels of the electron beams, the out‐of‐field dose generally decreased with an increase in the number of LA layers except for 6, 8, 10, 12, and 15 MeV at 5 and 8 cm from the CAX as a result of the buildup effect. Comparing a five‐layer LA with no LA for 4‐MeV electron beams, the single largest dose reduction was approximately 98% observed at 30 cm from the CAX. For 6‐, 8‐, 10‐, 12‐, and 15‐MeV electron beams and a distance of more than 8 cm from the CAX, the largest dose reduction was 95.0%, 92.2%, 87.3%, 85.6%, and 79.5%, respectively. Comparing a five‐layer LA with no LA for 6‐MeV beams, the dose reduction was 88.8%, 91.8%, 94.2%, 95.0%, 94.9%, and 90.5% at 13.5, 19, 24.5, 30, 41, and 63 cm from the CAX, respectively. In addition, the out‐of‐field dose reduction decreased with an increasing distance from the CAX, which was also observed for 8‐, 10‐, 12‐, and 15‐MeV beams. As illustrated in Figs. 3(g)–3(h), we also measured the peripheral dose in the cross‐plane direction (both sides of gantry: A side and B side) at a depth of 0.5 cm for an applicator size of 10 × 10‐cm^2^ and electron beam of 6 MeV. We observed that there was a similar effect on the LA shielding of a peripheral dose at both the cross‐plane and in‐plane.

### 3.C Shielding effect at 1.0‐cm depth

Figure 4 presents the peripheral dose profiles for different energy levels of electron beams and different distances from the CAX at a depth of 1 cm with a different number of LA layers. When the measured dose profiles were evaluated and normalized at Dref for different energy levels, it was observed that except for 4‐MeV electron beams and beyond 8 cm from the CAX, the peripheral doses decreased with an increasing distance from the CAX. For 6‐MeV electron beams, the peripheral dose reached 27.8%, 12.6%, 2.8%, 1.4%, 0.8%, 0.6%, and 0.3% of Dref at 5, 8, 13.5, 19, 24.5, 30, 41, and 63 cm from the CAX, respectively, when the number of LA layers gradually increased from zero (no LA) to five layers. For all electron beam energies at 1.0‐cm depth, there were no significant differences in the LA shielding effect when there were more than three LA layers. When the electron beam energy was >10 MeV, there were no noticeable changes in the LA shielding effect beyond 19 cm from the CAX.

### 3.D Shielding effect at 2.0‐cm depth

For different electron beam energies, the peripheral dose profiles were measured at a depth of 2.0 cm for a different number of LA layers (Fig. 5). When the electron beam energy was less than 10 MeV and there was more than one LA layer, the out‐of‐field dose value exhibited no obvious change beyond 8 cm from the CAX. For 6‐MeV electron beams, the out‐of‐field relative dose reached 4.0%, 2.0%, 1.6%, 1.5%, 1.3%, and 1.3%, respectively, with an increase in the number of LA layers 19 cm from the CAX. When the electron beam energy level was above 10 MeV, the peripheral dose exhibited no significant decrease after shielding with a different number of LA layers. For 12‐MeV electron beams, the peripheral relative dose was 1.1%, 0.7%, 0.6%, 0.5%, 0.4%, and 0.4% as the number of layers of the LA increased 19 cm away from the CAX.

### 3.E Shielding effect of peripheral dose for 6 × 6, 14 × 14, and 20 × 20‐cm^2^ applicators at 0.5‐cm depth

**FIG. 6:**
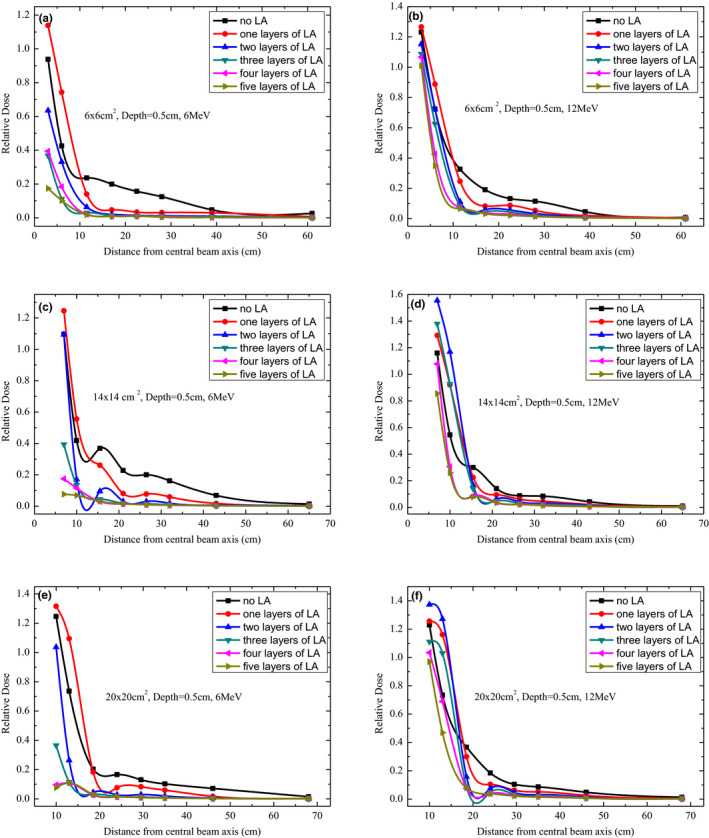
Out‐of‐field dose profiles for 6 and 12 MeV at a depth of 0.5 cm under different layers of lead aprons using 6 × 6, 14 × 14, and 20 × 20 cm^2^ applicators.

Figure 6 presents the profiles of the peripheral dose for electron beams of 6 and 12 MeV at a depth of 0.5 cm using 6 × 6, 14 × 14, and 20 × 20‐cm^2^ applicators. The dose profiles were normalized to Dref. As illustrated in Figs. 3, 4, 5, 6, the peripheral dose shielding effect of the LA was similar for applicators of different sizes of the linac. There was a buildup effect at a shorter distance from the applicator edge (<8 cm). Beyond 3 cm from the applicator edge, the peripheral dose decreased with an increase in the number of LA layers; however, there was no obvious difference in the peripheral dose shielding effect between four and five LA layers for different applicator sizes. For different electron beam energies and applicator sizes, the peripheral dose decreased with an increase in the distance from the applicator edge.

## 4 DISCUSSION

A megavoltage electron beam is an important treatment strategy in modern radiotherapy that is preferred due to its dosimetric characteristics and low penetration depth.^6^ The main source of an out‐of‐field dose contains photon and electron components. Bremsstrahlung photons may be produced when high‐energy electron beams, such as 15‐MeV beams, collide with the applicator building. The other component is electrons, which can occur by (a) scattering out, (b) penetrating through the applicator collimator building, or (c) leaking directly into the air from scattering foil without interacting with the applicator.^7^, ^8^, ^9^, ^10^, ^11^ For high‐energy electron beams, the out‐of‐field electron component is usually produced primarily via (2) and (3). For lower‐energy electron beams, scatter radiation occurs.^12^, ^13^ For 4‐MeV electron beams, the out‐of‐field dose peak may be caused by scattering electrons originating from the rounded surface of the MLC.^14^ According to our results, the peripheral dose peak gradually weakened with an increasing number of LA layers, which was likely caused by the low penetration depth of the electron beams.

The peripheral doses were considered harmful for patients and had to be minimized, especially when they were high. Out‐of‐field doses of the surface had a reduction of up to approximately 100% with LA shielding. According to our observations (Figs. 2, 3, 4, 5, 6), the LA shielding effect for lower‐energy electron beams was stronger than that for higher‐energy electron beams, which indicates that the LA was more effective at shielding an out‐of‐field dose of lower‐energy electron beams. For all energies and depths, there were no significant differences in the LA shielding effect between four and five layers of the LA; therefore, a four‐layer LA was sufficient for shielding a peripheral dose of 4–15‐MeV electron beams. However, the lowest value of the out‐of‐field dose did not reach zero, which may be attributed to a small number of photons caused by interactions with the scattering foil, applicator, and so on. Furthermore, according to our results, the effect of LA shielding was better for superficial peripheral radiation doses than for deep peripheral radiation doses. With an increasing distance from the CAX, the out‐of‐field doses gradually reduced, and the effect of LA shielding also declined. It was observed that out‐of‐field doses at large distances from the edge of the treatment field were mainly attributed to leakage radiation.^15^

The biological effect of radiation mainly consists of a deterministic effect and nondeterministic (stochastic) effect. The deterministic effect has a threshold dose, below which the effects are unobservable. However, there is no threshold dose for the nondeterministic effect. Nevertheless, the possibility of biological effects, such as cataracts, aplastic anemia, and secondary cancer,^16^ has a positive correlation with the amount of absorbed dose. Although doses outside the treatment field are small, they may have a significant radiobiological effect on OARs. Therefore, it is of great importance to protect patients from unnecessary radiation. This study suggests that out‐of‐field doses (beyond 3 cm from the applicator edge) from electron beams can be effectively shielded by adding several layers of an LA with 0.5 mm thickness on the surface of a water‐equivalent slab phantom. But LA should selectively be implemented within a distance of 3 cm from the applicator field because of the buildup effect. Shielding out‐of‐field doses thus provides significant overall protection for out‐of‐target volume beyond 3 cm from the applicator edge.

## 5 CONCLUSION

In this study, we successfully evaluated the shielding effect of an LA on out‐of‐field radiation doses for an Elekta Synergy linac using 6 × 6, 10 × 10, 14 × 14, and 20 × 20‐cm^2^ applicators. We determined that the shielding effect of an LA depends on the number of layers as well as the depth and distance away from the CAX. In this study, the maximum radiation dose reduction was almost 100% after LA shielding. In addition, the results demonstrated that four LA layers were sufficient for shielding out‐of‐field radiation (beyond 3 cm from the applicator edge) from 4 to 15 to MeV electron beams. Measuring the shielding effect of an LA can benefit radiation oncologists to protect patients from unnecessary radiation exposure.

## CONFLICT OF INTEREST

No conflict of interest.
